# Role of Nuclear Factor of Activated T Cells (NFAT) Pathway in Regulating Autophagy and Inflammation in Retinal Pigment Epithelial Cells

**DOI:** 10.3390/ijms22168684

**Published:** 2021-08-12

**Authors:** Hsuan-Yeh Pan, Ashley V. Ladd, Manas R. Biswal, Mallika Valapala

**Affiliations:** 1 School of Optometry, Indiana University, Bloomington, IN 47405, USA; hsupan@iu.edu (H.-Y.P.); ashladd@iu.edu (A.V.L.); 2 Department of Pharmaceutical Sciences, Taneja College of Pharmacy, University of South Florida, Tampa, FL 33612, USA; biswal@usf.edu; 3 Department of Ophthalmology, Morsani College of Medicine, University of South Florida, Tampa, FL 33612, USA; 4 Department of Internal Medicine, Morsani College of Medicine, University of South Florida, Tampa, FL 33612, USA; 5 Department of Molecular Genetics & Microbiology, College of Medicine, University of Florida, Gainesville, FL 32610, USA

**Keywords:** autophagy, lysosomal function, transcription factor EB, nuclear factor of activated in T cells (NFAT), calcineurin, retinal pigment epithelium (RPE)

## Abstract

Nuclear factor of activated T cells (NFAT) family of transcription factors are substrates of calcineurin and play an important role in integrating Ca^2+^ signaling with a variety of cellular functions. Of the five NFAT proteins (NFAT1-5), NFAT1-4 are subject to dephosphorylation and activation by calcineurin, a Ca^2+^-calmodulin-dependent phosphatase. Increased levels of intracellular Ca^2+^ activates calcineurin, which in turn dephosphorylates and promotes nuclear translocation of NFAT. We investigated the functions of NFAT proteins in the retinal pigment epithelial cells (RPE). Our results show that NFAT-mediated luciferase activity was induced upon treatment with the bacterial endotoxin, lipopolysaccharide (LPS) and treatment with the NFAT peptide inhibitor, MAGPHPVIVITGPHEE (VIVIT) decreased LPS-induced NFAT luciferase activity. LPS-induced activation of NFAT-regulated cytokines (IL-6 and IL-8) is inhibited by treatment of cells with VIVIT. We also investigated the effects of NFAT signaling on the autophagy pathway. Our results show that inhibition of NFAT with VIVIT in cells deprived of nutrients resulted in cytosolic retention of transcription Factor EB (TFEB), decreased expression of TFEB-regulated coordinated Lysosomal Expression and Regulation CLEAR network genes and decreased starvation-induced autophagy flux in the RPE cells. In summary, these studies suggest that the NFAT pathway plays an important role in the regulation of autophagy and inflammation in the RPE.

## 1. Introduction

Intracellular Calcium (Ca^2+^) regulates a wide variety of cellular functions via activation of diverse downstream cellular mediators. Increased intracellular levels of Ca^2+^ result in the activation of a Ca^2+^ and calmodulin-dependent phosphatase, calcineurin [1]. Calcineurin dephosphorylates and activates the nuclear factor of activated T cells (NFAT) family of transcription factors that possess critical roles in immune function, inflammatory responses, angiogenesis, cellular proliferation and differentiation [1,2,3,4]. The NFAT family consists of five members, NFAT1–NFAT5, except for NFAT5 all other members of the family are regulated by changes in intracellular Ca^2+^ [5]. NAFT5 is identified to transcriptionally induce a variety of genes associated with cellular response to osmotic stress [5]. All members of the NFAT family possess a conserved DNA-binding domain that shows structural similarity to the Rel-family proteins [6]. NFAT proteins possess a conserved *N*-terminal domain that contains multiple serine residues which are phosphorylated in resting conditions [6,7]. Nuclear translocation of NFAT proteins is inhibited upon phosphorylation in the N-terminal regulatory domain [6,7]. When cells are stimulated, de-phosphorylation of serine residues is mediated by the Ca^2+^-dependent phosphatase, calcineurin [6,7]. Dephosphorylation exposes the nuclear localization signal and promotes nuclear translocation and transcriptional induction of NFAT-regulated genes [6,7]. In response to Ca^2+^-mediated activation by calcineurin, NFAT proteins can either induce gene expression on their own or co-operatively with other transcription factors. NFAT proteins are known to associate with and function co-operatively with other transcription factors like Activator protein 1 (AP-1) to induce gene transcription [8]. Because of its interaction with other transcription factors, NFAT proteins can integrate signals from diverse pathways and perform a variety of cellular functions. One of the important functions of NFAT proteins in the immune cells is induction of inflammatory cytokine expression. The co-operative activity of NFAT and AP1 (Fos and Jun) proteins is important for the expression of few interleukins such as interleukin-2 (IL-2), IL-3, and IL-4 in T cells [8]. Calcineurin inhibitors, FK506 (tacrolimus) and cyclosporine A (CsA) are major immunosuppressants that bind to and inhibit the activity of calcineurin thereby dampening the transcriptional activity of NFAT [9]. NFAT was first identified to promote transcriptional induction of IL-2 in activated T cells and hence they are referred to as nuclear factors of activated T cells [10]. Contrary to their name, several studies have shown that NFAT proteins are expressed in a wide variety of cells and perform several non-immune functions [11,12,13]. Here, we investigated the expression of NFAT proteins and their functions in the RPE. Since, NFAT1-4 proteins are regulated by inflammatory-meditators, we tested whether these proteins are involved in inflammatory responses in the RPE. In addition to elucidating the role of the NFAT signaling pathway in regulating inflammatory responses, we also investigated the previously unknown functions of this pathway in regulating the process of autophagy. Nutrient deprivation is known to result in mucolipin-mediated lysosomal Ca^2+^ release causing activation of calcineurin, subsequent dephosphorylation and nuclear translocation of transcription Factor EB (TFEB) [14]. TFEB is a major transcription factor associated with concerted activation of both lysosomal and autophagy genes in a majority of cell types. TFEB regulates a wide variety of genes belonging to the coordinated lysosomal expression and regulation (CLEAR) network by binding to an E-box-like response element [15]. The CLEAR network genes are composed of autophagy and lysosomal genes and under conditions of stress, activation of CLEAR network genes results in concerted activation of autophagy and lysosomal pathways [16]. The activation and nuclear translocation of TFEB is regulated by phosphorylation. Under normal conditions, TFEB is phosphorylated by mammalian target of rapamycin complex 1 (mTORC1) and retained in the cytosol [17]. During stress, TFEB is de-phosphorylated by calcineurin and translocated to the nucleus to activate gene expression [14]. Both TFEB and NFAT are dephosphorylated and activated by calcineurin in response to stimuli that cause an increase in cytosolic Ca^2+^. Recent studies have suggested that nutrient deprivation induces calcineurin-dependent nuclear translocation of both TFEB and NFAT [14].

In this study, we investigated the previously unidentified role of NFAT in the RPE. Since, NFAT is a major transcription factor involved in the regulation of inflammatory cytokines, we investigated the role of NFAT pathway in regulating cytokine expression in the RPE. Additionally, both NFAT and TFEB proteins are known to be translocated to the nucleus in response to starvation in a calcineurin-dependent manner [14], hence, we investigated the role of the NFAT pathway in regulating starvation-induced autophagy responses the RPE.

## 2. Results

### 2.1. Expression of NFAT in the RPE

**Figure 1:**
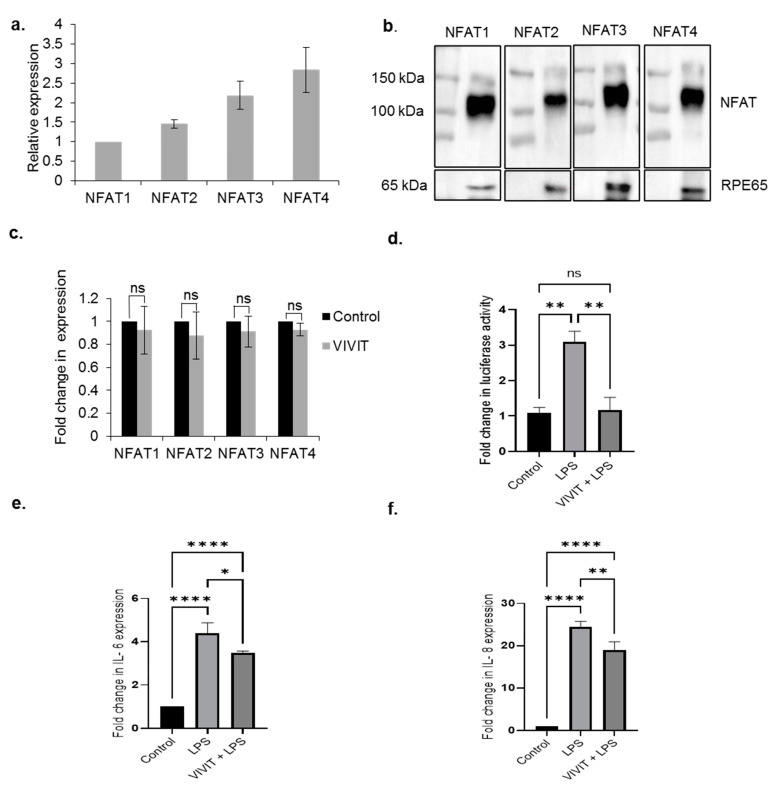
Expression of NFAT proteins in the RPE. (**a**) qRT-PCR analysis of the expression of NFAT isoforms in ARPE-19 cells. (**b**) Immunoblot analysis of the expression of NFAT isoforms, NFAT1-4 in isolated mouse RPE extracts. (**c**) Expression analysis of NFAT1-4 in ARPE-19 cells treated with 20 µM VIVIT for 48 h. Student’s *t*-test (two tailed) was used for statistical analysis. (**d**) Luciferase activity in AD-NFAT-luciferase vector transduced ARPE-19 cells treated with 10 µM VIVT for 4 h followed by treatment with 10 µg/mL LPS for 24 h. (**e**) qRT-PCR analysis of expression of IL-6 in ARPE-19 cells treated with 10 µM VIVIT followed by treatment with 10 µg/mL LPS for 24 h. (**f**) qRT-PCR analysis of expression of IL-8 in ARPE-19 cells treated with 10 µM VIVIT followed by treatment with 10 µg/mL LPS for 24 h. Values represent mean ± s.d. of at least three independent experiments. One-way ANOVA was used for analysis *p*-value. The *p*-value is stated as * *p* < 0.05, ** *p* < 0.01, **** *p* < 0.0001.

We first investigated the expression of NFAT isoforms in the RPE. Our results show that ARPE-19 cells express all the NFAT isoforms, NFAT1-4. Of the NFAT isoforms, the expression levels of NFAT1 and NFAT2 are lower compared to NFAT3 and NFAT4 (Figure 1a). Our immunoblotting studies also show expression of NFAT1-4 isoforms in isolated mouse RPE extracts (Figure 1b). Next, we used a cell permeable peptide inhibitor of NFAT, VIVIT that inhibits calcineurin-mediated dephosphorylation and nuclear translocation of NFAT [18]. We studied whether treatment with VIVIT resulted in changes in the levels of NFAT mRNA. Our studies revealed that VIVIT did not result in any changes in the cellular expression of NFAT mRNA (Figure 1c).

### 2.2. LPS Induces the Expression of NFAT-Regulated Inflammatory Cytokines in the RPE

Since, NFAT proteins are regarded as transcription factors that induce the expression of inflammatory cytokines [1], we studied NFAT-regulated inflammatory network in the RPE. We analyzed whether treatment with the bacterial endotoxin lipopolysaccharide (LPS) results in NFAT activation by performing a luciferase reporter assay using an adenoviral NFAT luciferase reporter vector (AD-NFAT-luc). The AD-NFAT-luc vector contains four tandem repeats of NFAT binding site which control the expression of luciferase. Our results show about a 3-fold induction of luciferase activity (*p* ≤ 0.01) in cells treated with 10 µg/mL LPS for 24 h. Treatment of AD-NFAT-luc-transduced cells with 10 µM VIVT for 4 h followed by treatment with 10 µg/mL LPS for 24 h resulted in significant (2.66-fold) reduction of LPS-induced luciferase activity (*p* ≤ 0.01) in ARPE-19 cells (Figure 1d). These results suggest that in RPE cells, LPS-induced transcriptional activity of NFAT is inhibited by treatment with the NFAT peptide inhibitor, VIVIT [18]. We next evaluated whether transcriptional activation of NFAT in the presence of LPS is associated with upregulation of the expression of NFAT-regulated cytokines in ARPE-19 cells. qRT-PCR analysis showed a 4.39-fold increase (*p* ≤ 0.0001) in the expression of IL-6 in cells treated with 10 µg/mL LPS for 24 h. Pre-treatment of cells with the NFAT peptide inhibitor, VIVIT (10 µM VIVT for 6 h) followed by LPS treatment (10 µg/mL for 24 h) resulted in a 1.26-fold decrease (*p* ≤ 0.05) in the expression of IL-6 compared to cells treated with LPS only (Figure 1e). We also investigated the expression of IL-8, another potent inflammatory cytokine in the RPE. qRT-PCR analysis revealed a 24-fold (*p* ≤ 0.0001) in the expression of IL-8 upon treatment with 10 µg/mL for 24 h. Pre-treatment of ARPE-19 cells with VIVIT followed by treatment with LPS (10 µg/mL for 24 h) results in a 1.3-fold reduction (*p* ≤ 0.01) in the expression of IL-8 (Figure 1f). These results suggest that inhibition of NFAT activation by VIVIT causes a significant reduction in the expression of inflammatory cytokines in ARPE-19 cells.

### 2.3. Involvement of NFAT Signaling in the Autophagy Pathway

**Figure 2:**
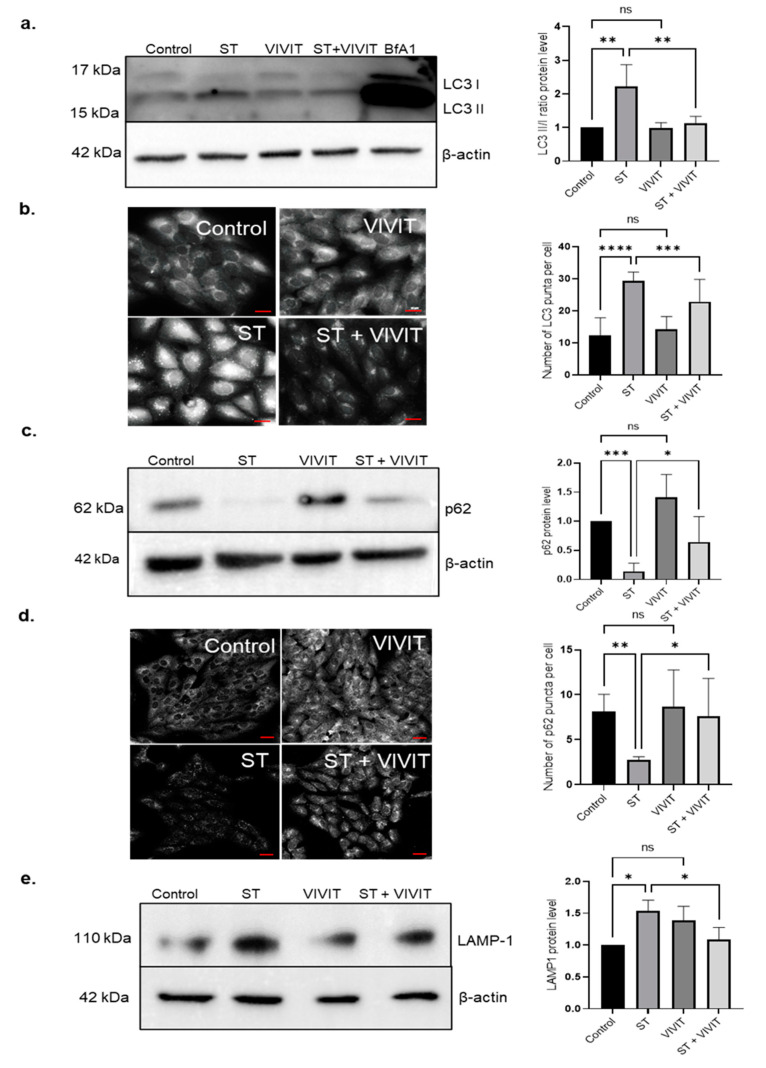
Inhibition of NFAT downregulates autophagy flux and lysosomal function. (**a**) Immunoblot analysis of LC3 expression in ARPE-19 cells subjected to nutrient deprivation and treatment with 20 µM VIVIT for 48 h. (**b**) The number of LC3 puncta was quantified by LC3 immunostaining of cells treated with 20 µM VIVIT and subjected to nutrient deprivation for 48 h. (**c**) Immunoblot analysis to determine the expression of p62 in ARPE-19 cells treated with 20 µM VIVIT and subjected to nutrient deprivation for 48 h. (**d**) Immunostaining with p62 antibody to determine cellular levels of p62 in ARPE-19 cells treated with 20 µM VIVIT and subjected to nutrient deprivation for 48 h. (**e**) Immunoblot analysis to measure the expression of LAMP-1 in ARPE-19 cells treated with 20 µM VIVIT and subjected to nutrient deprivation for 48 h. Values represent mean ± s.d. of at least three independent experiments. One-way ANOVA was used for analysis *p*-value. The *p*-value is stated as * *p* < 0.05, ** *p* < 0.01, *** *p* < 0.001, **** *p* < 0.0001. LC3 image scale = 20 μm, p62 image scale = 100 µm. (ST indicates starvation, BfA1 indicates Bafilomycin A1).

It is known that NFAT proteins are dephosphorylated and activated by a phosphatase, calcineurin [9]. In addition to regulating the activity of NFAT proteins, calcineurin is also known to regulate de-phosphorylation and activation of a master controller of autophagy, transcription factor EB (TFEB) [14]. TFEB functions as a master regulator of this transcription program by controlling the expression of genes involved in lysosome biogenesis and function and autophagy [19]. Our previous studies have shown that the expression of TFEB is induced in ARPE-19 cells and primary mouse RPE subjected to nutrient deprivation [20]. We also previously showed that overexpression of constitutively active TFEB induces the expression of autophagy and lysosomal genes in the RPE [20]. Since both TFEB and NFAT are regulated by calcineurin, we investigated whether inhibition of NFAT signaling by VIVIT affects the autophagy pathway in cells subjected to nutrient deprivation. We measured autophagy flux in cells treated with the inhibitor VIVIT, and in cells subjected to nutrient deprivation. Nutrient deprivation resulted in a significant increase in an LC3II/LC3I ratio, showing increased flux through the autophagy pathway in response to starvation. In cells co-treated with VIVIT and subjected to nutrient deprivation, we observed a 2-fold decrease (*p* ≤ 0.01) in the LC3II/LC3I ratio compared to cells subjected to nutrient deprivation alone. We did not observe any significant changes in the LC3II/LC3I ratio in cells treated with VIVIT only compared to control cells. Autophagy flux was also monitored by treating the cells with the lysosomal inhibitor, Bafilomycin A1. A significant increase in the levels of LC3II was observed in cells treated with Bafilomycin A1 indicating accumulated LC3II as a result of lysosomal inhibition (Figure 2a). Immunostaining with anti-LC3 antibody also showed a significant increase in the number of LC3 puncta in nutrient-deprived cells. Quantification of LC3 puncta revealed a 1.3-fold decrease (*p* ≤ 0.001) in LC3 puncta in cells treated with VIVIT and subjected to nutrient deprivation compared to cells treated with starvation alone. However, no significant difference was observed in the LC3 puncta in cells treated with VIVIT only compared to untreated control cells (Figure 2b). Since our results showed a reduction in autophagy flux in cells co-treated with VIVIT and subjected to starvation, we evaluated the expression of p62, an autophagy substrate in these cells. Nutrient deprivation resulted in a decrease in p62 in ARPE-19 cells. Starved cells treated with VIVIT showed a 4.65-fold increase (*p* ≤ 0.05) in the accumulation of p62 compared to cells subjected to starvation alone, suggesting that VIVIT inhibits starvation-induced degradation of p62 (Figure 2c). p62 immunostaining showed a 2.8-fold increase (*p* ≤ 0.05) in the cellular levels of p62 in cells treated with VIVIT and starvation compared to cells subjected to starvation alone (Figure 2d). It is known that lysosomes are crucially important for the completion of the autophagy pathway [21]. Induction of the autophagy pathway is always accompanied by a concomitant induction of both lysosomal number and function. We measured cellular levels of the lysosomal maker, LAMP-1 in the presence of VIVIT. Our results show a 1.4-fold decrease (*p* ≤ 0.05) in the expression of endogenous LAMP-1 in cells treated with VIVIT and subjected to starvation compared to cells subjected to starvation alone (Figure 2e).

### 2.4. NFAT-Mediated Regulation of TFEB Expression and Activity

**Figure 3:**
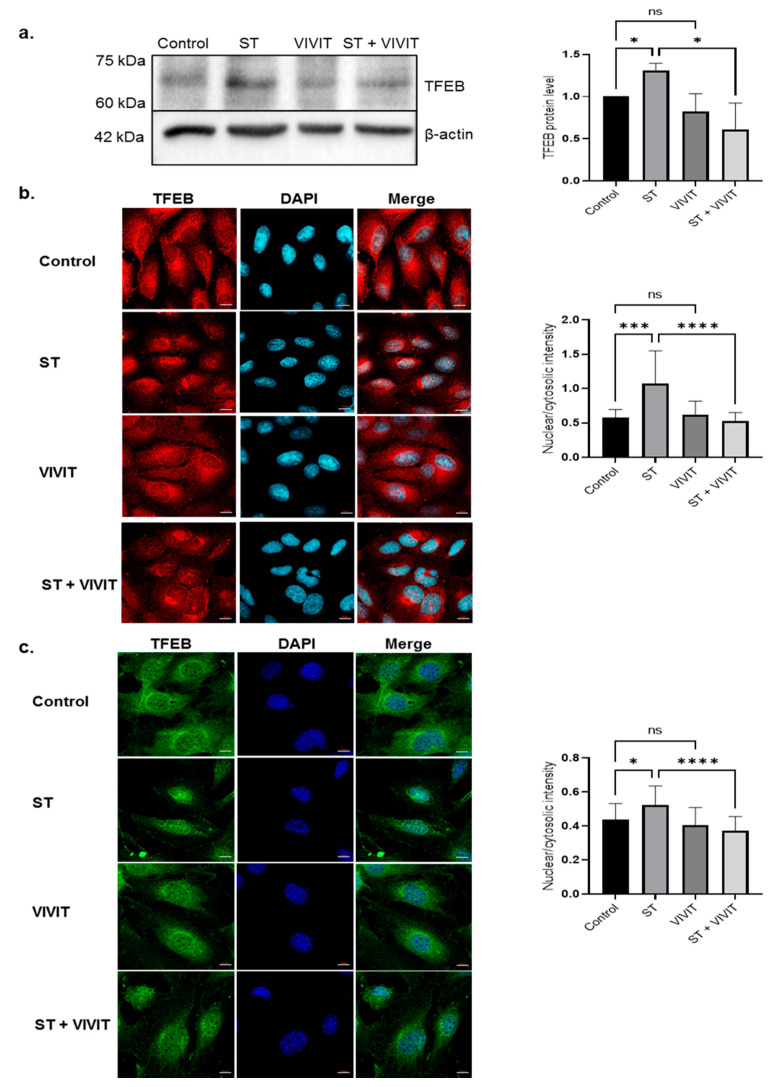
NFAT-mediated regulation of TFEB expression and activity. (**a**) Immunoblot analysis of expression of TFEB in ARPE-19 cells treated 20 µM VIVIT and subjected to 48 h period of nutrient deprivation. (**b**) Nuclear and cytosolic localization of TFEB in ARPE-19 was examined by immunostaining with TFEB antibody. (**c**) Nuclear and cytosolic localization of TFEB in HPV E6/E7 immortalized mouse RPE cells subjected to starvation and VIVIT treatment. Values represent mean ± s.d. of at least three independent experiments. One-way ANOVA was used for analysis *p*-value. The *p*-value is stated as * *p* < 0.05, *** *p* < 0.001, **** *p* < 0.0001. Scale = 10 μm (ST indicates starvation).

Since inhibition of NFAT signaling dampened starvation-induced autophagy responses, we investigated whether NFAT inhibition has any effect on the TFEB pathway. Immunoblotting studies revealed a 2.1-fold decrease (*p* ≤ 0.05) in starvation-induced expression of TFEB in cells subjected to starvation and VIVIT treatment compared to cells subjected to nutrient deprivation alone. ARPE-19 cells treated with VIVIT alone did not show any changes in the expression of TFEB compared to control (Figure 3a). We also observed a predominant nuclear localization of TFEB in cells subjected to nutrient deprivation. However, in cells subjected to starvation and VIVIT treatment, TFEB expression was predominantly confined to the cytosol. Cytosolic localization of TFEB in cells treated with VIVIT alone was comparable to that of control cells (Figure 3b). We also investigated subcellular localization of TFEB in mouse RPE cells immortalized using human papilloma virus (HPV) 16 (E6/E7). These cells are previously shown to possess phenotypic characteristics of RPE cells in vivo [22]. Our studies showed cytosolic retention of starvation-induced TFEB in transformed mouse RPE cells subjected to treatment with VIVIT. Nuclear localization of TFEB was observed under starvation and TFEB was confined to the cytosol in control and VIVIT-treated cells (Figure 3c).

### 2.5. Effects of NFAT Inhibition on the Expression of TFEB-Regulated Genes

**Figure 4:**
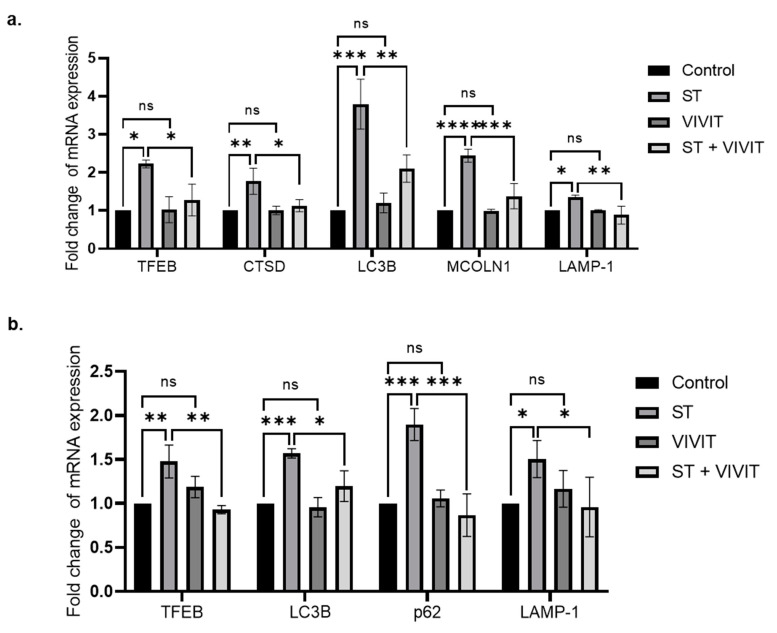
NFAT-mediated regulation of TFEB expression and CLEAR network genes. (**a**) qRT-PCR analysis of the expression of TFEB and TFEB downstream targets: lysosomal genes (CTSD, LAMP-1 and MCOLN1) and autophagy genes (MAP1LC3B) upon VIVIT treatment (20 µM) in ARPE-19 cells subjected to nutrient deprivation for 48 h. (**b**) qRT-PCR analysis of the expression of TFEB and TFEB downstream targets: lysosomal (LAMP-1) and autophagy genes (MAP1LC3B and p62) upon starvation and VIVIT (20 µM) treatment for 48 h in HPV E6/E7 immortalized mouse RPE cells. Values represent mean ± s.d. of at least three independent experiments. One-Way ANOVA was used for analysis *p*-value. The *p*-value is stated as * *p* < 0.05, ** *p* < 0.01, *** *p* < 0.001, **** *p* < 0.0001. (ST indicates starvation).

Next, we analyzed the expression of TFEB by qRT-PCR analysis, our results show that treatment of ARPE-19 cells with VIVIT by itself did not result in any change in the expression levels of TFEB mRNA. Expression of TFEB mRNA was significantly induced by 2.2-fold (*p* ≤ 0.05) in cells subject to nutrient deprivation compared to control cells. A 1.7-fold (*p* ≤ 0.05) decrease in starvation-induced expression of TFEB in cells subjected to starvation and VIVIT treatment was observed compared to cells treated with starvation alone. We also investigated the expression of TFEB-regulated CLEAR network genes and our results show that the expression of MAP1LC3B (LC3B) was decreased by 1.8-fold (*p* ≤ 0.01) in cells treated with VIVIT and subjected to nutrient deprivation compared to cells treated with starvation alone. VIVIT by itself did not result in any changes in the expression of LC3B compared to control cells. In addition, we investigated the expression of other CLEAR network genes involved in autophagy and lysosomal pathways, we observed that the expression of Cathepsin D (CTSD), lysosomal associated membrane protein 1 (LAMP-1), mucolipin-1 (MCOLN1) were decreased by 1.6- (*p* ≤ 0.05), 1.5- (*p* ≤ 0.01) and 1.8-fold (*p* ≤ 0.001), respectively in cells treated with VIVIT and nutrient-deprivation compared with cells subjected to nutrient-deprivation alone (Figure 4a). We also investigated the expression of TFEB-regulated CLEAR network genes in transformed mouse RPE cells. Our results show that starvation by itself increased the expression of TFEB (1.47-fold (*p* ≤ 0.01)) and the following TFEB-regulated CLEAR network genes: LC3 (1.57-fold (*p* ≤ 0.001)), p62 (1.89-fold (*p* ≤ 0.001)) and LAMP-1 (1.5-fold (*p* ≤ 0.05)) (Figure 4b). On the other hand, starvation-induced expression of TFEB was decreased by 1.58-fold (*p* ≤ 0.01) in cells subjected to starvation and VIVIT treatment. The expression of TFEB-regulated CLEAR network genes, LC3B, p62, and LAMP-1 were decreased by 1.32-fold (*p* ≤ 0.05), 2.19-fold (*p* ≤ 0.001) and 1.57-fold (*p* ≤ 0.05), respectively (Figure 4b) in cells subjected to starvation and VIVIT treatment compared to cells treated with starvation alone.

### 2.6. Effects of NFAT Inhibition on mTOR and AKT Signaling Pathways

**Figure 5:**
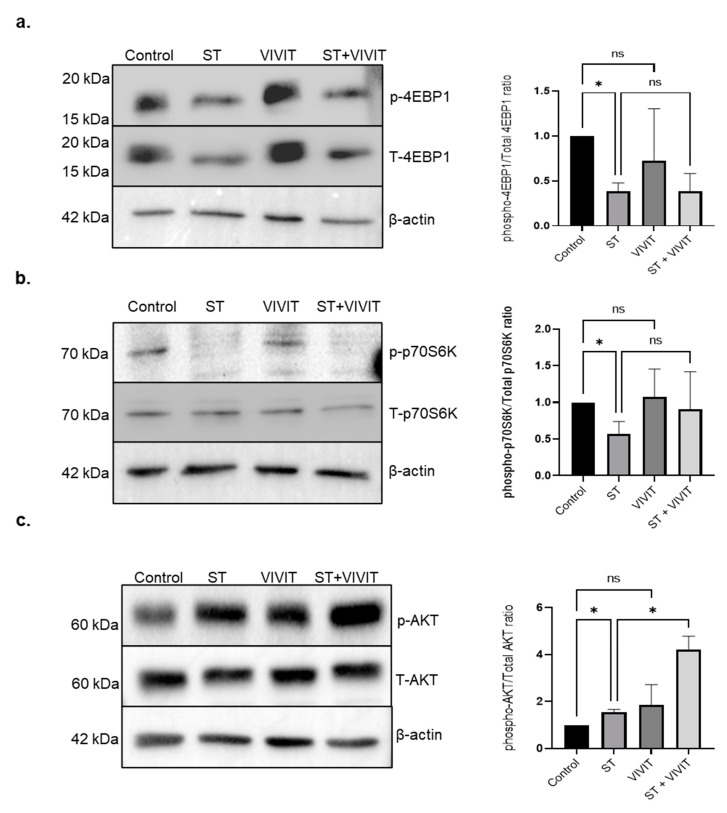
Effects of NFAT inhibition on mTOR and AKT signaling pathway. (**a**,**b**) Immunoblot analysis of expression of p-4EBP-1 and p-p70S6K in ARPE-19 cells upon treatment with 20 µM VIVIT in starved cells for 48 h. (**c**) Immunoblot analysis of expression of p-AKT in ARPE-19 cells treated with 20 µM VIVIT and subjected to nutrient deprivation for 48 h. Values represent mean ± s.d. of at least three independent experiments. One-way ANOVA was used for analysis *p*-value. The *p*-value is stated as * *p* < 0.05. (ST indicates starvation).

Since, NFAT inhibition resulted in a retention of TFEB in the cytosol upon nutrient deprivation, we investigated whether inhibition of NFAT effects key signaling pathways that regulate subcellular localization of TEFB. Our studies show that the levels of mTOR substrates, p-4EBP-1 and p-p70S6K were decreased in starved cells due to inhibition of mTOR pathway upon nutrient stress. The expression of p-4EBP-1 and p-p70S6K upon inhibition of NFAT in starved cells was not altered compared to cells subjected to starvation alone. These results suggest that inhibition of NFAT does not alter mTOR signaling pathway in RPE cells (Figure 5a,b). Next, we investigated whether NFAT inhibition affects the AKT signaling pathway, which is also known to influence the subcellular localization of TFEB [23]. Our results show that inhibition of the NFAT pathway in starved cells resulted in a 2.72-fold induction (*p* ≤ 0.05) in the expression of p-AKT compared to cells subjected to nutrient deprivation alone (Figure 5c).

## 3. Discussion

In this study, we investigated the role of NFAT signaling pathway in the regulation of LPS-induced cytokine expression and the autophagy pathway in ARPE-19 cells. We observed that treatment with LPS significantly induced NFAT luciferase activity. LPS-induced cytokine expression was decreased upon inhibition of NFAT activity using a peptide inhibitor, VIVIT which inhibits the activation of NFAT by calcineurin. Our results clearly show that NFAT inhibition by VIVIT decreases expression of LPS-induced IL-6 and IL-8 in ARPE-19 cells. NFAT signaling pathway is widely studied in immune cells and is targeted for the treatment of several autoimmune diseases [24]. Immunosuppressive drugs known to inhibit calcineurin include cyclosporin A (CsA) and FK506 [25]. These inhibitors bind to intracellular proteins to form complexes that directly bind to and inhibit the phosphatase activity of calcineurin and thereby inhibiting the activity of NFAT [26]. Since calcineurin regulates several biological processes, inhibiting the activity of calcineurin can potentially affect other signaling pathways in the cell. In this study, we used a cell permeable peptide inhibitor, VIVIT, that was designed to interfere with calcineurin binding to NFAT and selectively inhibit calcineurin-mediated NFAT activation without effecting the activity of calcineurin [27]. Previous studies have shown that VIVIT is based on the PxIxIT consensus sequence, a conserved sequence in all NFAT members that corresponds to the calcineurin docking site in NFAT [27,28]. Recently, another consensus sequence was identified in the C-terminus of NFAT that is a docking site for calcineurn and is termed as the LxVP sequence located in the C-terminus of NFAT [29]. Studies have also shown that expression of the NFATc1-YLAVP peptide inhibited starvation-induced nuclear translocation of TFEB [30].

Since, NFAT and TFEB, a transcription factor known to play a fundamental role in autophagy and lysosomal function, are both activated by the Ca^2+^-regulated phosphatase, calcineurin [14], we evaluated the cellular effects of NFAT inhibition on the autophagy processes regulated by TFEB. Previous studies have also shown the involvement of NFAT signaling in phagocytic responses [31,32]. Inhibition of NFAT activity by calcineurin was shown to cause a delay in phagocytic activity of macrophages [33]. Although VIVIT does not inhibit the cellular activity of calcineurin, we investigated whether treatment with VIVIT affects the TFEB-regulated transcriptional program in the RPE. Our studies show that inhibition of NFAT in cells subjected to nutrient deprivation showed a decrease in the flux through the autophagy pathway and inhibits autophagic-degradation of p62 compared to cells subjected to nutrient deprivation alone. Our results show that in cells subjected to nutrient deprivation, treatment with VIVIT decreased expression of LAMP-1. The decrease in starvation-induced autophagy is also shown to be accompanied by cytosolic retention of TFEB and downregulation of genes in the CLEAR network. In addition, treatment with VIVIT also did not have any effect on the mTOR signaling pathway as indicated by the levels of mTOR downstream targets, 4EBP-1 and P70S6K which remained unchanged in cells treated with VIVIT compared to control cells.

Imbalances in autophagy and lysosomal function is implicated in the pathogeneses of age-related macular degeneration (AMD) [34,35]. Increased oxidative stress in the ageing RPE is known to result in RPE degeneration and accumulation of undigested cellular material as a result of reduced autophagy in these cells [36]. Restoring autophagy is known to decrease oxidative stress and prevent RPE degeneration [36,37]. TFEB regulates transcription of genes involved in the autophagy and lysosomal pathway [14]. Induction of TFEB expression was shown to promote clearance of pathological cellular substrates in several diseases [19,38]. In this study, we investigated the involvement of NFAT in TFEB-regulated responses in the RPE. Our studies demonstrate a previously unknown role of the NFAT signaling pathway in the regulation of TFEB transcriptional program in the RPE. We have shown that the NFAT pathway is involved in LPS-induced expression of cytokines and inhibition of NFAT with VIVIT effects autophagy pathway under conditions of nutrient deprivation. We also observed an induction in the levels of p-AKT in cells subjected to starvation and VIVIT treatment. We believe that increased p-AKT levels are responsible for cytosolic retention of TFEB in response to starvation and associated decrease in TFEB-regulated transcriptional program as shown in previous studies [20]. The mechanism by which NFAT inhibition increases the levels of p-AKT in starved cells and the involvement of the NFAT/TFEB axis in the regulation of autophagy in RPE cells in vivo is subject to further investigation.

## 4. Materials and Methods

### 4.1. Cell Culture, Animal Studies and Treatment

Adult Retinal Pigment Epithelial cell line-19 (ARPE-19) cells were kindly provided by Dr. James T. Handa, Wilmer Eye Institute, JHMI. The cells were cultured in Dulbecco’s Modified Eagle Medium: Nutrient Mixture F-12 (DMEM/F12) containing L-Glutamine and 15 mM HEPES (Gibco, Thermo Fisher Scientific) and supplemented with 10% Fetal Bovine Serum (Hyclone, GE Healthcare Life Sciences) and 1% Antibiotic-Antimitotic (Gibco, Thermo Fisher Scientific). The HPV E6/E7 immortalized mouse retinal pigmented epithelial Cell line HPV E6/E7 was obtained from (Applied Biological Materials Inc. # T0574) and cultured in Prigrow III medium supplemented with 10% Fetal Bovine Serum (Hyclone, GE Healthcare Life Sciences) and 1.5% Antibiotic-Antimitotic (Gibco, Thermo Fisher Scientific). ARPE-19 and HPV E6/E7 cells were maintained at 37 °C in a humidified incubator with an atmosphere of 5% CO2. ARPE-19 cells and immortalized mouse RPE cells were cultured in Earle’s Balanced salt solution (EBSS) (Gibco, Thermo Fisher Scientific) and treated with NFAT inhibitor, VIVIT, at 20 µM for 48 h. The protein was collected from the RPE tissue of C57BL/6J mice for immunoblotting as previously published [39]. The experimental procedures were approved by the Institutional Animal Care and Use Committee, Indiana University/School of Optometry and conformed to the ARVO Statement for the Use of Animals in Ophthalmologic and Vision Research.

### 4.2. Antibodies

All the primary antibodies are provided in Table S1.

### 4.3. Quantitative Real Time-PCR

APRE-19 and HPV E6/E7 immortalized mouse RPE cells were cultured in a 6-well plate to 65–70% confluency. The treatment was performed as described. Total RNA was extracted from APRE-19 and HPV E6/E7 immortalized mouse RPE cells by using the TRIzol reagent (Ambion, Life Technologies, Waltham, MA, USA) and reverse transcribed to cDNA by using the High-Capacity RNA to cDNA Kit (Appliedbiosystems, Thermo Fisher Scientific). Real-time PCR analysis was performed using SsoAdvanced Universal SYBR Green Supermix (Bio-Rad, Hercules, CA, USA) and TaqMan™ Fast Advanced Master Mix (Thermo Scientific). Expression of each gene were normalized to 18s and β-actin. Real-time PCR was performed on CFX96TM Real-Time System (Bio-Red). All the sequences of primers are provided in Table S2.

### 4.4. Immunoblotting

APRE-19 cells were cultured in a 6-well plate to 65–70% confluency. The treatment was performed as described. ARPE-19 cells were washed with ice-cold 1× PBS and lysed on ice by 1× RIPA buffer containing protease inhibitor cocktail (Cytoskeleton, Inc., Denver, CO, USA). Protein concentration was measured by BCA protein assay (Thermo Scientific). A total of 20–30 µg of lysate was resolved by SDS-PAGE gel and transferred onto PVDF membranes (Millipore sigma). Membranes were block with 5% milk in TBST at room temperature for 1 h and incubated with primary antibodies overnight at 4 °C. Secondary antibodies were used and incubated at room temperature for 1 h. The membranes were developed with the SuperSignal Chemiluminescent (Thermo Scientific) and scanned with Image Lab software (Bio-Rad Laboratories)

### 4.5. Immunostaining and Microscopy

ARPE-19 cells and immortalized mouse RPE cells were cultured in 8-well chamber slides to 55–60% confluency. The treatment was performed as described. The cells were fixed by 4% paraformaldehyde for 15 min at room temperature, and then permeabilized with 0.5% triton-x diluted in PBS for 15 min. The cells were blocked for 1 h with 5% BSA in 1× PBS containing 0.5% Tween-20 and 10% goat serum (MP Biomedicals, Santa Ana CA, USA). The primary antibodies were added and the cells at a concentration and incubated overnight at 4 °C. The following day, the cells were washed and added the secondary antibodies for 1 h at room temperature in the dark. The cells were mounted by ProLong Glass Antifade Mountant (Invitrogen, Thermo Fisher). The cells were observed by a Zeiss LSM800 microscope (20× and 63× objective). Images were quantified using the Image J software.

### 4.6. Luciferase Assay

ARPE-19 cells were cultured in 96-wells plates to 55–60% confluency and treated NFAT-adenovirus vector (Vector Biolabs, Malvern, PA USA) for 24 h. After 24 h, cells were pre-treated with 10 µM VIVIT (Calbiochem, San Diego, CA, USA, Millipore Sigma, Burlington, MA, USA) for 4 h and followed by treatment of LPS for 24 h. The cells were washed twice in PBS and lysed with 20 μL of 1× Lysis Buffer (Promega, Madison, WI, USA) for luciferase determination. NFAT luciferase activity was measured using the Luciferase Assay System (Promega). Luciferase activities were measured using Thermo Appliskan Multimode Microplate Reader (Thermo Fisher Scientific).

### 4.7. Quantification of Data and Statistical Analysis

The number of p62 and LC3 puncta were quantified using the default “Analyze Particles” plugin in Image J. The corrected total cell fluorescence (CTCF) was calculated by subtracting the background intensity from cells intensity using Image J. The intensity of Western blots was quantified using image J.

All data is represented as mean ± standard deviation (SD). The data from at least three independent experiments was used to obtain statistical significance. Student’s *t*-test (two tailed) was used to compare NFAT expression in VIVIT treated and control cells (Figure 1c). One-way ANOVA with Tukey post hoc multiple comparison was performed for other experiments. Statistical analyses were performed using GraphPad Prism software. A value of *p* ≤ 0.05 was considered as an indication of statistical significance. The *p*-value is stated as * *p* < 0.05, ** *p* < 0.01, *** *p* < 0.001, **** *p* < 0.0001.

## Data Availability

The datasets generated in the manuscript will be made available from the corresponding author on reasonable request.

## Supplementary Materials

The following are available online at https://www.mdpi.com/article/10.3390/ijms22168684/s1.
