# Supplementary material for: Role of Nuclear Factor of Activated T Cells (NFAT) Pathway in Regulating Autophagy and Inflammation in Retinal Pigment Epithelial Cells
Source: Int J Mol Sci. 2021 Aug 12;22(16):8684. doi: 10.3390/ijms22168684 (PMC8395439; doi:10.3390/ijms22168684)
Supplement: Supplementary file 1 [file ijms-22-08684-s001.zip › ijms-1282748-supplementary.pdf]

# Supplementary Material

**Table S1**

| **Antibodies** | **Application** | **Vendor** |
|---|---|---|
| Transcription factor EB | WB: 1:500 IF :100 | Proteintech |
| anti-LC3 antibody | WB: 1:1000 IF 1:500 | MBL International |
| anti-SQSTM1/p62 antibody | WB: 1:10000 IF: 1:400 | Abcam |
| anti-β-Actin antibody | WB: 1:2000 | Sigma |
| 4EBP-1 antibody | WB: 1:1000 | Cell signaling technology |
| Phospho-4EBP-1 antibody | WB: 1:1000 | Cell signaling technology |
| p70S6K antibody | WB: 1:1000 | Cell signaling technology |
| Phospho-p70S6K antibody | WB: 1:1000 | Cell signaling technology |
| AKT antibody | WB: 1:1000 | Cell signaling technology |
| Phospho-AKT antibody | WB: 1:1000 | Cell signaling technology |
| LAMP-1 antibody | WB: 1:1000 | Cell signaling technology |
| NFAT1 (Anti-NFATc2 Antibody) | WB: 1:200 | Santa Cruz Biotechnology,Inc. |
| NFAT2 (NFATC1 Monoclonal Antibody) | WB: 1:2,000 | Invitrogen (ThermoFisher Scientific) |
| NFAT3 (Anti-NFATc4 Antibody) | WB: 1:100 | Santa Cruz Biotechnology,Inc. |
| NFAT4 (Anti-NFATc3 Antibody) | WB: 1:200 | Santa Cruz Biotechnology,Inc. |
| RPE65 antibody | WB: 1:100 | Santa Cruz Biotechnology,Inc. |

**Table S2**

| **Human Primer** | **Sequence** |
|---|---|
| TFEB-R | CGTCCAGACGCATAATGTTGTC |
| TFEB-F | ACCTGTCCGAGACCTATGGG |
| LAMP-1-R | TGTTCTCGTCCAGCAGACAC |
| LAMP-1-F | CTGCCTTTAAAGCTGCCAAC |
| CTSD-R | CCCGAGCCATAGTGGATGT |
| CTSD-F | CACCACAAGTACAACAGCGAC |
| MCON1-R | AACTCGTTCTGCAGCAGGAAGC |
| MCON1-F | TCTTCCAGCACGGAGACAAC |
| LC3-R | AGATTGGTGTGGAGACGCTG |
| LC3-F | AGCAGCTTCCTGTTCTGGAT |
| 18s-R | AGTCGGCATCGTTTATGGTC |
| 18s-F | CGCGGTTCTATTTTGTTGGT |
| IL-6-R | GTCAGGGGTGGTTATTGCAT |
| IL-6-F | GGAGACTTGCCTGGTGAAAA |
| IL-8-R | GGTGGAAAGGTTTGGAGTATGTCTT |
| IL-8-F | CTTCCTGATTTCTGCAGCTCTGT |
| NFAT1-R | CGTTTTCTCTTCCCATTGATGAC |
| NFAT1-F | AAGAGCCAGCCCAACATGC |
| NFAT2-R | ACTGACGTGAACGGGGCTGG |
| NFAT2-F | CTGTGCAAGCCGAATTCTCTGG |
| NFAT3-R | AGCGTCACCTCGTTGCTCTGC |
| NFAT3-F | GTCCTGATGGGAAGCTGCAATGG |
| NFAT4-R | TGATGTGGTAAGCAAAGTGGTGTGGT |
| NFAT4-F | GCGGCCTGCAGATCTTGAGC |
| Mouse Primer | Sequence |
| β-actin-R | ACCAGAGGCATACAGGGACA |
| β-actin-F | CTAAGGCCAACCGTGAAAAG |
| P62-R | GCCAAAGTGTCCATGTTTCA |
| P62-F | AGGGAACACAGCAAGCT |
| β-actin | Mm02619580_g1 Taqman probe |
| TFEB | Mm00448968_m1 Taqman probe |
| LC3 | Mm00782868_sH Taqman probe |
| LAMP-1 | Mm00495262_m1 Taqman probe |
